# Advancing adaptation of evidence-based interventions through implementation science: progress and opportunities

**DOI:** 10.3389/frhs.2023.1204138

**Published:** 2023-06-05

**Authors:** David A. Chambers

**Affiliations:** Division of Cancer Control and Population Sciences, National Cancer Institute (NIH), Bethesda, MD, United States

**Keywords:** adaptation, evidence-based interventions, implementation science, implementation, dynamism

## Abstract

While the recognition of the need to adapt interventions to improve their fit with populations and service systems has been well established within the scientific community, limited consideration of the role of adaptation within implementation science has impeded progress toward optimal uptake of evidence-based care. This article reflects on the traditional paths through which adapted interventions were studies, progress made in recent years toward better integration of the science of adaptation within implementation studies with reference to a special publication series, and next steps for the field to continue to build a robust knowledge base on adaptation.

## Introduction

With the many advantages of the evidence-based medicine movement (1), now well into its third decade, there have been limitations in the inherent value placed on the fidelity to manualized interventions as the main driver to improvement of clinical and community practice. While there are strengths of adhering to interventions that have been rigorously tested and demonstrate beneficial health outcomes when intervention integrity is maintained, there has long been evidence of mismatches between the design and their ability to be implemented for all who could benefit.

## Considering adaptation as a pre-condition to implementation

Until the last decade or so, the primary route to advancing the science of adaptation was to consider the reformulation of the intervention to better fit with a specific population or delivery setting and then to test that intervention in a new clinical trial, either against a control intervention or again care as usual. If the adapted intervention was shown to be beneficial, it would then be “ready for implementation” with the expectation that this new form of the intervention be adhered to as designed and tested.

There were several consequences of this approach to advancing a science of adaptation. First, the potential for infinite permutations of each intervention arose, as one could justifiably argue that adaptation could be needed based on multiple demographic and contextual variables, and each would thus require its own new clinical trial. Second, the adapted interventions could be just as inflexible in their formulation as the parent intervention, so that any additional mismatches identified in their implementation would require a return to the adaptation and testing cycle. Third, less thought was given to the core elements of an intervention that would ensure health benefit; if the “intervention package” was always being tested fully, little information could be gleaned about what intervention elements were universal and what elements would need tailoring. Perhaps most important, the role of patients, clinicians, communities, and settings in shaping ongoing adaptation to interventions was largely non-existent. The science of adaptation was significantly limited.

## Progress in understanding adaptation in the context of implementation

In recent years, great progress has been made in building out this area within implementation science. Through conceptual advances like the Dynamic Adaptation Process (2), several iterations of the Framework for Reporting Adaptations and Modifications to Evidence-based Interventions (FRAME) (3, 4), and systematic reviews of adaptation research (5, 6), we have seen new recognition of the complexity and nuance of improving the fit between interventions and service delivery settings, Collectively, these efforts have recognized the importance of ongoing adaptation of interventions during implementation due to dynamic settings and needs, the distinction between the form and function (7) of interventions influencing what should be constant or variable in an intervention's delivery, the potential for ongoing learning about adaptation throughout the implementation process, and the development of a taxonomy of adaptations to guide both adaptation research and practice. Indeed, the vision of an “adaptome” where evidence on intervention adaptation could be amassed into an accessible store of knowledge for use by the field is significantly closer (8). This collection of papers on adaptations of interventions speaks to what is possible when we move beyond the traditional paradigm to a new focus on iterative learning during evidence-based practice implementation.

In considering the articles in this series, a number of major themes emerge that speaks to the progress in the past few years. First, the elaboration of frameworks that are inclusive of adaptation (9–11) has provided conceptual guidance and support in enabling the operationalization of adaptation types, as well as identifying key determinants of effective intervention tailoring. Second, the articles demonstrate that intervention adaptations occur throughout the implementation process, from exploration all the way to sustainment (12). Third, effective adaptation requires key partners (e.g., patients, clinicians, community members, administrators, policymakers) to help identify when and how intervention adaptation is needed, the utility of interventions for matching need, and approaches to improve the fit between the supply of interventions and the demand for them. Finally, there is an increase in the volume of empirical data on adaptation for a variety of different interventions utilized in a range of different service settings over time.

## Discussion

With appreciation for the investigators participating in this series of articles and many more in our field working on the adaptation of evidence-based interventions, we can now contemplate exciting new directions that will further extend the science. First, for the most part, our evidence on adaptations of evidence-based interventions come from individual research studies. This may limit the full range of evidence we can collect, namely missing out on “practice-based evidence” as tailoring of interventions to settings and populations occurs frequently outside of studies. Opportunities to build an ongoing learning system on adaptation so that we can move from considering individual adaptations to ongoing evolution of interventions could significantly benefit our implementation efforts (8).

Relatedly, much of our work on adaptations has been reactive; we see a mismatch between implementation and context and refine to address it. Moving more toward a proactive view towards adaptation will encourage us to expect these mismatches going forward and plan for them. Adapting and tailoring to context has been identified as a category of implementation strategy and yet it seems to be less frequently used as we study the range of approaches to support uptake of evidence-based interventions. Planning for adaptation and measuring the impact of those adaptations in the spirit of a learning healthcare system could be of great benefit going forward (12).

Finally, we can continue to improve the design of our health interventions, more clearly defining core components that are empirically supported as immutable and (as CFIR has long suggested) the “adaptable periphery” which encourages ongoing adaptation as needed (9). Imagine if each evidence-based intervention had clear specifications for both core and adaptable elements. Given the competing demands and multiple challenges that our health and community systems face each day, any new intervention will need to co-exist with what is already being delivered, and improvement of “fit” (13) may go a long way towards enhancing equitable implementation so that evidence-based care is accessible for all. This is, of course, related to ongoing work to distinguish between the “form” and “function” of interventions (7), and would assist us in moving beyond the dichotomy between fidelity and adaptation; the optimum lies in between.

## Data Availability

The original contributions presented in the study are included in the article, further inquiries can be directed to the corresponding author.
